# Elevated Paracellular Glucose Flux across Cystic Fibrosis Airway Epithelial Monolayers Is an Important Factor for *Pseudomonas aeruginosa* Growth

**DOI:** 10.1371/journal.pone.0076283

**Published:** 2013-10-04

**Authors:** James P. Garnett, Michael A. Gray, Robert Tarran, Malcolm Brodlie, Christopher Ward, Emma H. Baker, Deborah L. Baines

**Affiliations:** 1 Division of Biomedical Sciences, St George's University of London, London, United Kingdom; 2 Institute for Cell and Molecular Biosciences, Newcastle University, Newcastle-upon-Tyne, United Kingdom; 3 Cystic Fibrosis/Pulmonary Research and Treatment Centre, University of North Carolina, Chapel Hill, North Carolina, United States of America; 4 Institute of Cellular Medicine, Newcastle University, Newcastle-upon-Tyne, United Kingdom; University of Pittsburgh, School of Medicine, United States of America

## Abstract

People with cystic fibrosis (CF) who develop related diabetes (CFRD) have accelerated pulmonary decline, increased infection with antibiotic-resistant *Pseudomonas aeruginosa* and increased pulmonary exacerbations. We have previously shown that glucose concentrations are elevated in airway surface liquid (ASL) of people with CF, particularly in those with CFRD. We therefore explored the hypotheses that glucose homeostasis is altered in CF airway epithelia and that elevation of glucose flux into ASL drives increased bacterial growth, with an effect over and above other cystic fibrosis transmembrane conductance regulator (CFTR)-related ASL abnormalities. The aim of this study was to compare the mechanisms governing airway glucose homeostasis in CF and non-CF primary human bronchial epithelial (HBE) monolayers, under normal conditions and in the presence of *Ps. aeruginosa* filtrate. HBE-bacterial co-cultures were performed in the presence of 5 mM or 15 mM basolateral glucose to investigate how changes in blood glucose, such as those seen in CFRD, affects luminal *Ps. aeruginosa* growth. Calu-3 cell monolayers were used to evaluate the potential importance of glucose on *Ps. aeruginosa* growth, in comparison to other hallmarks of the CF ASL, namely mucus hyperviscosity and impaired CFTR-dependent fluid secretions. We show that elevation of basolateral glucose promotes the apical growth of *Ps. aeruginosa* on CF airway epithelial monolayers more than non-CF monolayers. *Ps. aeruginosa* secretions elicited more glucose flux across CF airway epithelial monolayers compared to non-CF monolayers which we propose increases glucose availability in ASL for bacterial growth. In addition, elevating basolateral glucose increased *Ps. aeruginosa* growth over and above any CFTR-dependent effects and the presence or absence of mucus in Calu-3 airway epithelia-bacteria co-cultures. Together these studies highlight the importance of glucose as an additional factor in promoting *Ps. aeruginosa* growth and respiratory infection in CF disease.

## Introduction

The airway epithelium is covered by a thin layer of surface liquid (ASL), which is vital for maintaining a healthy respiratory tract. It is now well established in cystic fibrosis (CF) that a lack of functional cystic fibrosis transmembrane conductance regulator (CFTR) impairs airway bicarbonate and fluid secretion producing an acidic, viscous ASL [1], [2], [3] that is readily colonised by bacteria such as *Pseudomonas aeruginosa* [4]. Much has been made of the defective anti-microbial properties of the CF ASL and of changes in host-pathogen responses across the CF airway epithelium. However, relatively little has been reported on disease-related changes in the nutritional content of ASL, such as elevated glucose concentrations, that could stimulate bacterial growth in CF airways.

In people with CF, coexisting diabetes mellitus (CF-related diabetes; CFRD) is associated with an increased risk of infection with multiple antibiotic-resistant *Ps. aeruginosa*. Patients with CFRD have more pulmonary exacerbations, which are less likely to respond to intravenous antibiotics, than those without diabetes [5]. We have previously shown that ASL glucose concentrations are elevated in patients with CF (∼2 mM; compared to ∼0.4 mM in healthy individuals) and are further elevated in CFRD to ∼4 mM [6]. In intubated patients on intensive care, we found that elevated glucose concentrations in bronchial aspirates were associated with the presence and acquisition of respiratory pathogens [7]. In mice, glucose concentrations were elevated in lung secretions from diabetic compared to wild-type animals and bacterial load was greater in the airways of diabetic than wild-type mice [8], [9]. In human airway epithelial cells grown at air-liquid interface, elevation of basolateral glucose concentrations increased ASL glucose concentrations and directly promoted the growth of respiratory pathogens such as *Ps. aeruginosa* [8].

ASL glucose concentration is the net effect of the paracellular diffusion of glucose from the blood/interstitial fluid across the airway epithelium into ASL and its removal from ASL by epithelial uptake through facilitative glucose transporters, GLUT2 and GLUT10 [9], [10], [11], [12]. We have previously shown that paracellular glucose diffusion is increased not only by elevating basolateral glucose concentrations (e.g. hyperglycaemia), but also by inflammation, which disrupts tight junction integrity. In human epithelial cell monolayers, we found that application of either apical pro-inflammatory cytokines [10] or apical bacterial pathogens [8] increased paracellular glucose flux, increasing glucose concentrations in ASL. Under pro-inflammatory conditions, airway epithelial glucose uptake through apical GLUTs is elevated, but this is insufficient to compensate for the increased paracellular glucose leak and does not prevent elevation of ASL glucose concentrations [10]. Control of paracellular glucose movement thus appears to be the main rate-limiting step for maintaining low ASL glucose concentrations. Since the CF airway epithelium is known to be more susceptible to inflammation-induced changes in tight junctions and paracellular permeability compared to non-CF epithelium [13], we hypothesised that this may account for the elevation in ASL glucose concentrations observed in CF patients without diabetes, which is augmented further by hyperglycaemia in CFRD.

The aim of this study was to compare the mechanisms governing airway glucose homeostasis in polarized monolayers of CF and non-CF primary human bronchial epithelial (HBE) cells, under normal conditions and in the presence of *Ps. aeruginosa* filtrate. HBE-bacterial co-cultures were performed in the presence of 5 mM or 15 mM basolateral glucose to investigate how changes in glucose concentration, such as those seen in CFRD, affect apical *Ps. aeruginosa* growth. In addition, Calu-3 human airway epithelial cell monolayers were used to evaluate the potential importance of glucose on *Ps. aeruginosa* growth, in comparison to other hallmarks of the CF ASL, namely mucus hyperviscosity and impaired cystic fibrosis transmembrane conductance regulator (CFTR)-dependent fluid secretions.

## Methods

### Primary Human Bronchial Epithelial Cell Culture

Primary human bronchial epithelial (HBE) cells (non-CF and CF) were obtained from endobronchial brushings or extracted from explanted lungs and cultured as previously described [14], [15], [16]. HBE cells were obtained in accordance with approval from the relevant ethics committees and informed written consent from all study patients: Newcastle and North Tyneside Local Regional Ethics Committee (reference number 2001/179 and 07/Q0906/47); The University of North Carolina at Chapel Hill Biomedical Institutional Review Board (protocol #03-1396). Cells were transferred onto clear Costar Transwell® inserts (0.45-µm pore size) and grown at air-liquid-interface (ALI) to form confluent monolayers. Cells were studied 3–5 weeks post-seeding.

### Calu-3 Cell Culture

The human adenocarcinoma-derived cell line, Calu-3 (from ATCC), was grown in Eagle's minimal essential medium (EMEM) plus 10% FCS, 2 mM L-glutamine, 100 units/ml penicillin, 100 µg/ml streptomycin, and 1% non-essential amino acids (Sigma) and incubated in humidified air containing 5% CO_2_ at 37°C.

Calu-3 cells were seeded onto clear Costar Transwell® inserts (0.45-µm pore size) at 250,000 cells/cm^2^ to form confluent polarised monolayers, as previously described [17]. Experiments were carried out 10–14 days post-seeding.

For studies examining the effect of a viscous mucus layer on bacterial growth in co-culture, Calu-3 monolayers were grown at ALI 5 days post-seeding on transwell supports. Under these conditions a thick mucus layer builds up on the apical surface which can be removed by washing the surface with PBS. 72 hours before the experiment, transwells were washed to remove the mucus layer and placed in Krebs salt solution consisting of 115 mM NaCl, 5 mM KCl, 25 mM NaHCO_3_, 1 mM MgCl_2_, 1 mM CaCl_2_ and 5 mM D-glucose (equilibrated with 5% CO_2_ to pH 7.4) to remove antibiotics and components of the media that could influence bacterial growth. The cells were incubated to allow the mucus layer to re-form. The transwells were then divided into two parallel sets. One set of transwells were re-washed to remove this layer just prior to experimentation, while another set were lightly washed to preserve the mucus layer.

### Ps. aeruginosa Culture

*Ps. aeruginosa* strain PA01 was used for the experiments. A single colony of PA01 was incubated overnight at 37°C in EMEM (containing 5 mM glucose) to form a culture of approximately 5×10^9^ colony forming units (CFU)/ml.

*Ps. aeruginosa* filtrate was prepared by boiling (10 minutes), centrifuging (5,500× G for 30 minutes) and then filtering (0.45 µm pore filter) the overnight bacterial culture, as previously described [18]. The filtrate was diluted 1∶10 in glucose-free media prior to addition to airway epithelial cell cultures. It has previously been shown that 10% *Ps. aeruginosa* filtrate is not highly cytotoxic to HBE cells over a similar time course [19].

### Paracellular L-glucose flux and D-glucose uptake experiments

Paracellular movement of glucose across HBE monolayers was measured by analysis of radiolabelled [^14^C]-L-glucose transepithelial flux. Experiments were initiated by adding 1 ml of Krebs salt solution containing 1.0 µCi of [^14^C]-l-glucose plus 10 mM of non-radiolabelled equivalent glucose to the basolateral side of the transwells and 0.1 ml of glucose-free Krebs salt solution to the apical side. Apical and basolateral samples were taken after 1 h and the concentration of radiolabelled glucose was analysed using a scintillation counter.

[14C]-D-glucose uptake experiments on HBE monolayers were performed as previously described [10].

### *In vitro* co-culture model

Bacterial cultures were diluted in glucose-free Krebs salt solution (composition described above). 50 µl or 100 µl of 1×10^7^ CFU/ml PA01 was applied to the apical surface of HBE or Calu-3 cell monolayers, respectively. The basolateral side of the airway epithelial-bacterial co-cultures were washed and placed in Krebs salt solution 24 hours prior to co-culture experiments to remove antibiotics and components of the media that could influence bacterial growth. Normal blood glucose or hyperglycaemia was modelled by applying basolateral glucose concentrations of 5 and 15 mM, respectively. Co-cultures were placed in a CO_2_ incubator at 37°C for 7 hours, after which each was homogenised and CFU calculated by plating out serial dilutions.

### ASL glucose

To evaluate the effects of *Ps. aeruginosa* and/or basolateral glucose on the glucose concentration of the ASL overlying Calu-3 monolayers, the apical surface was washed and then 100 µl of glucose-free Krebs salt solution was placed onto the surface of monolayers with or without *Ps. aeruginosa* filtrate. The basolateral side was supplied with Krebs salt solution containing either 5 or 15 mM D-glucose. Cultures were left for 24 hours to equilibrate. Apical fluid was subsequently removed from the cell surface and glucose concentrations were measured using a glucose oxidase analyser (Analox Instruments, UK).

### Transepithelial Liquid Secretion

The rate and pH of liquid secreted from Calu-3 monolayers was measured as previously described [17], after 7 hours of co-culture with PA01.

### Chemicals and reagents

All chemicals and reagents were obtained from Sigma, Poole, UK unless otherwise stated.

### Statistical analysis

Values are reported as mean ± S.E.M. Statistical analysis was performed using analysis of variance (ANOVA) and post hoc Bonferroni multiple comparison or Student's *t* test. *P* values <0.05 were considered significant.

## Results

### CF airway epithelial monolayers exhibit similar rates of glucose uptake to non-CF monolayers

**Figure 1:**
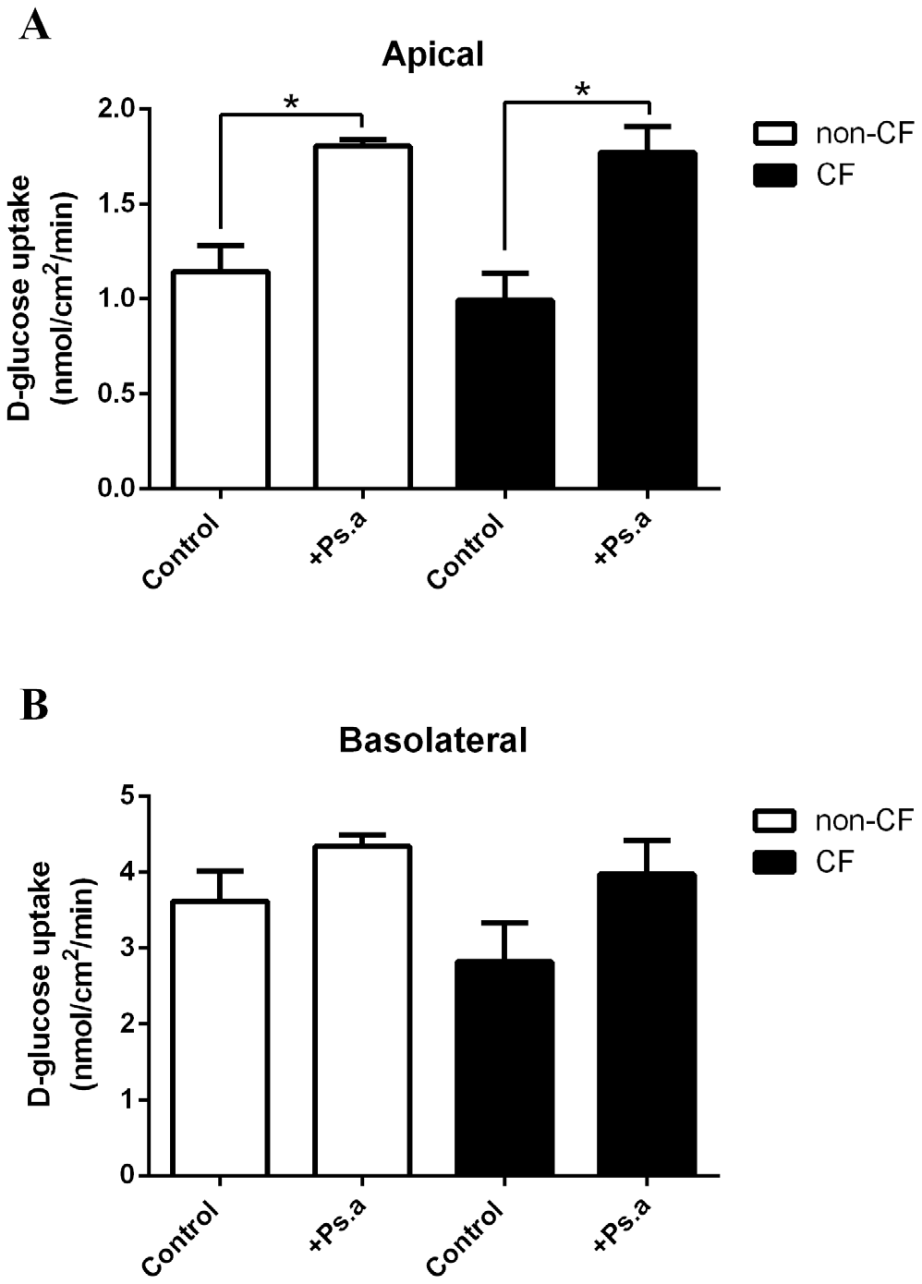
*Ps. aeruginosa* filtrate increases apical glucose uptake across non-CF and CF human airway epithelial monolayers. Glucose (^14^C-D-glucose) uptake across the apical (**A**) and basolateral (**B**) membrane of non-CF and CF HBE monolayers, in the presence and absence of *Ps. aeruginosa* filtrate (Ps. a). n = 3 in each group, obtained using cells from 3 different non-CF and CF donors assayed in 3 separate experiments. * p<0.05.

Comparison of ^14^C-D-glucose uptake rates by non-CF and CF human bronchial epithelial (HBE) monolayers showed no significant difference in either apical or basolateral uptake (Figure 1). Apical glucose uptake was 1.1±0.1 nmol/cm^2^/min and 1.0±0.1 nmol/cm^2^/min across non-CF and CF HBE monolayers, respectively (p>0.05, n = 3; Figure 1A). Basolateral glucose was significantly higher than apical uptake in both cell types: 3.6±0.2 nmol/cm^2^/min and 2.9±0.3 nmol/cm^2^/min across non-CF and CF HBE monolayers, respectively (n = 3; Figure 1B).

### *Ps. aeruginosa* filtrate increases apical glucose uptake across human airway epithelial monolayers

The addition of *Ps. aeruginosa* filtrate to the apical surface (for 24 hours) led to an increase in apical, but not basolateral, D-glucose uptake by non-CF (58±12% increase; p<0.05, n = 3; Figure 1A) and CF (78±14%; p<0.05, n = 3) HBE monolayers. No significant difference in *Ps. aeruginosa*-induced glucose uptake was observed between non-CF and CF HBE monolayers (p>0.05, n = 3).

### *Ps. aeruginosa* filtrate reduces transepithelial resistance and enhances paracellular glucose flux across CF epithelium to a greater extent than non-CF epithelium

**Figure 2:**
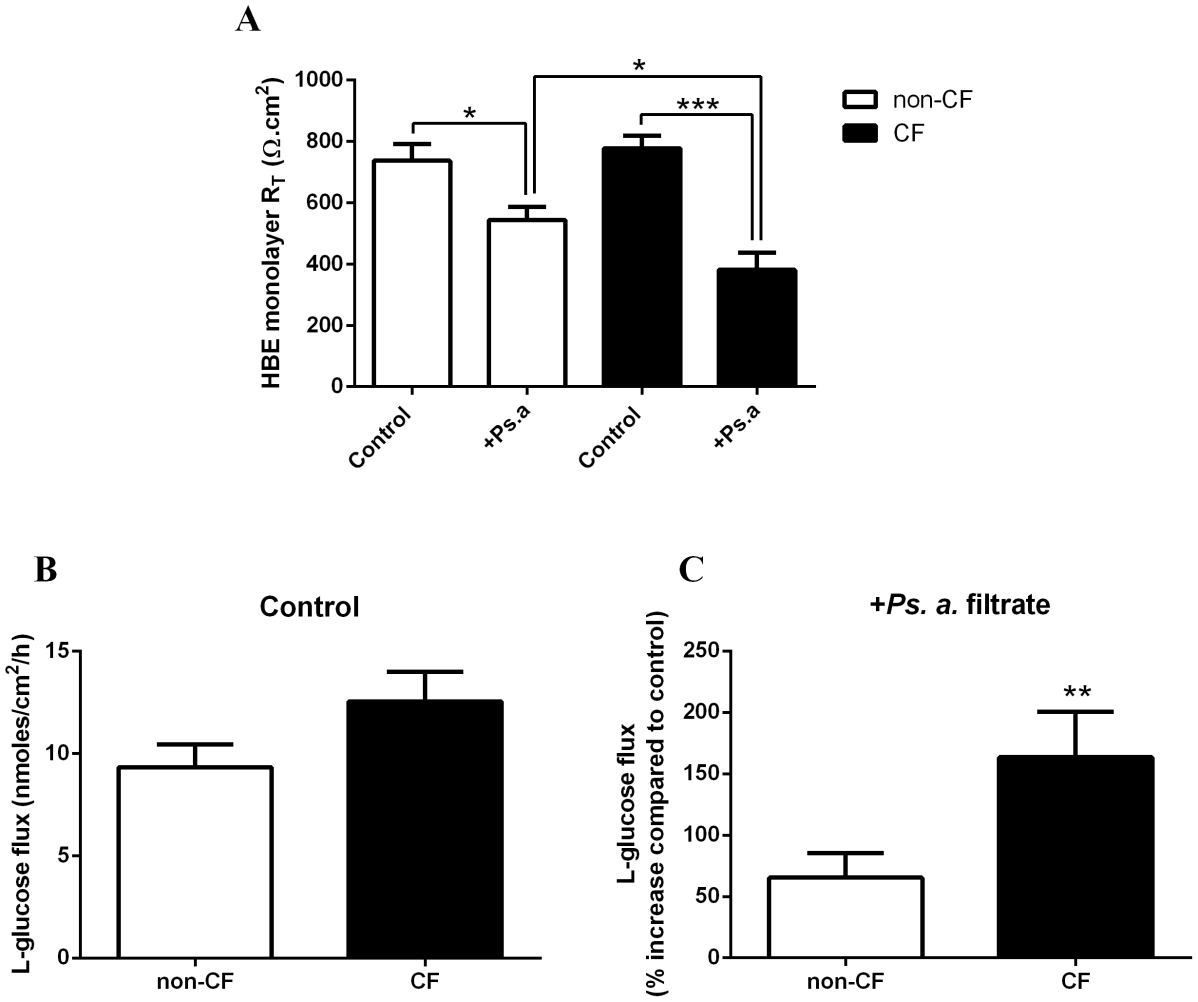
*Ps. aeruginosa* filtrate reduces transepithelial resistance and enhances paracellular glucose flux across CF epithelium to a greater extent than non-CF epithelium. A: Transepithelial resistance (R_T_) across non-CF and CF HBE monolayers, in the presence and absence of *Ps. aeruginosa* filtrate (Ps. a). n = 6 in each group, obtained using cells from 3 different non-CF and CF donors assayed in 2 separate experiments. * p<0.05, *** p<0.001. B: Basolateral-to-apical paracellular L-glucose (^14^C-L-glucose) flux across untreated non-CF and CF HBE monolayers. n = 4 in each group, obtained using cells from 2 different non-CF and CF donors assayed across 4 separate experiments. C: Basolateral-to-apical paracellular L-glucose (^14^C-L-glucose) flux across non-CF and CF HBE monolayers in the presence of *Ps. aeruginosa* filtrate (Ps. a). n = 4 in each group, obtained using cells from 2 different non-CF and CF donors assayed across 4 separate experiments. ** p<0.01.

Comparison of the transepithelial resistance (R_T_; Figure 2A) and the paracellular basolateral-to-apical flux of L-glucose (Figure 2B) across non-CF and CF HBE monolayers showed no significant difference between the cell types under control conditions (p>0.05, n = 3). However, addition of the bacterial filtrate to the apical surface of HBE monolayers (for 24 hours) produced a significant reduction in R_T_ and a corresponding increase in the paracellular flux of glucose. *Ps. aeruginosa* filtrate induced a significantly larger decrease in R_T_ across CF HBE monolayers (778±41 Ω.cm^2^ to 381±56 Ω.cm^2^; p<0.05, n = 6; Figure 2A) compared to non-CF HBE monolayers (737±55 Ω.cm^2^ to 544±42 Ω.cm^2^; p<0.001, n = 6). Similarly, the *Ps. aeruginosa*-induced increase in paracellular L-glucose flux was much greater across CF HBE monolayers (164±19%; p<0.01, n = 4; Figure 2C) compared to non-CF HBE monolayers (65±10%, p<0.05, n = 4).

### Elevating basolateral D-glucose concentration increases the growth of apical *Ps. aeruginosa* across airway epithelial monolayers

**Figure 3:**
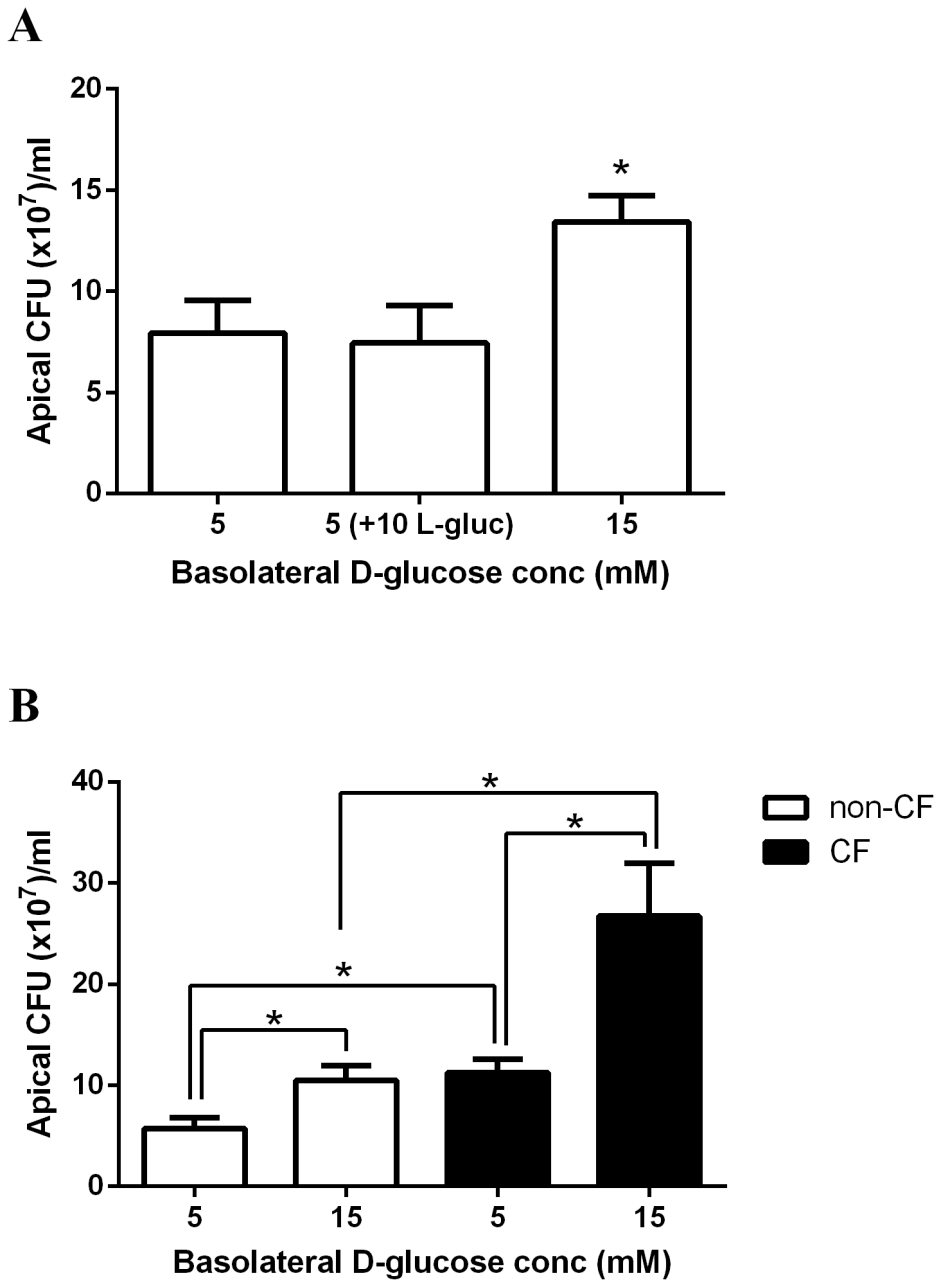
Basolateral glucose-induced growth of apical *Ps. aeruginosa* is greater across CF airway epithelial monolayers. **A**: *Ps. aeruginosa* growth over 7 hours across the apical surface of HBE monolayers, in the presence of 5 mM D-glucose, 15 mM basolateral D-glucose or 5 mM D-glucose plus 10 mM L-glucose. n = 4 in each group, obtained using cells from 2 different non-CF and CF donors assayed across 4 separate experiments. * p<0.05. **B**: *Ps. aeruginosa* growth over 7 hours across the apical surface of non-CF and CF HBE monolayers, in the presence of 5 mM or 15 mM basolateral D-glucose. n = 4 in each group, obtained using cells from 2 different non-CF and CF donors assayed across 4 separate experiments. * p<0.05.

*Ps. aeruginosa* was added to the apical surface of non-CF HBE monolayers for 7 hours in the presence of 5 mM or 15 mM basolateral D-glucose. Increasing the basolateral concentration of D-glucose from 5 to 15 mM resulted in a 69±13% increase in apical *Ps. aeruginosa* growth (p<0.05, n = 4; Figure 3A). Increasing the L-glucose, rather than D-glucose, concentration in the basolateral solution did not enhance apical bacterial growth.

### Basolateral glucose-induced growth of apical *Ps. aeruginosa* is greater across CF airway epithelial monolayers

Interestingly, although elevation of basolateral glucose from 5 to 15 mM enhanced apical bacterial growth across both non-CF and CF HBE monolayers, the glucose-induced increase in *Ps. aeruginosa* growth was greater across CF HBE monolayers (p<0.05, n = 4; Figure 3B). Raising basolateral glucose increased the apical concentration of *Ps. aeruginosa* from 5.8±1.0 CFU (x10^7^)/ml to 10.5±1.4 CFU (×10^7^)/ml and from 11.3±1.3 CFU (x10^7^)/ml to 26.8±5.2 CFU (x10^7^)/ml, on non-CF and CF HBE monolayers, respectively.

### Apical *Ps. aeruginosa* addition increases glucose flux across Calu-3 airway epithelial monolayers, resulting in elevated ASL glucose and D-glucose dependent bacterial growth

**Figure 4:**
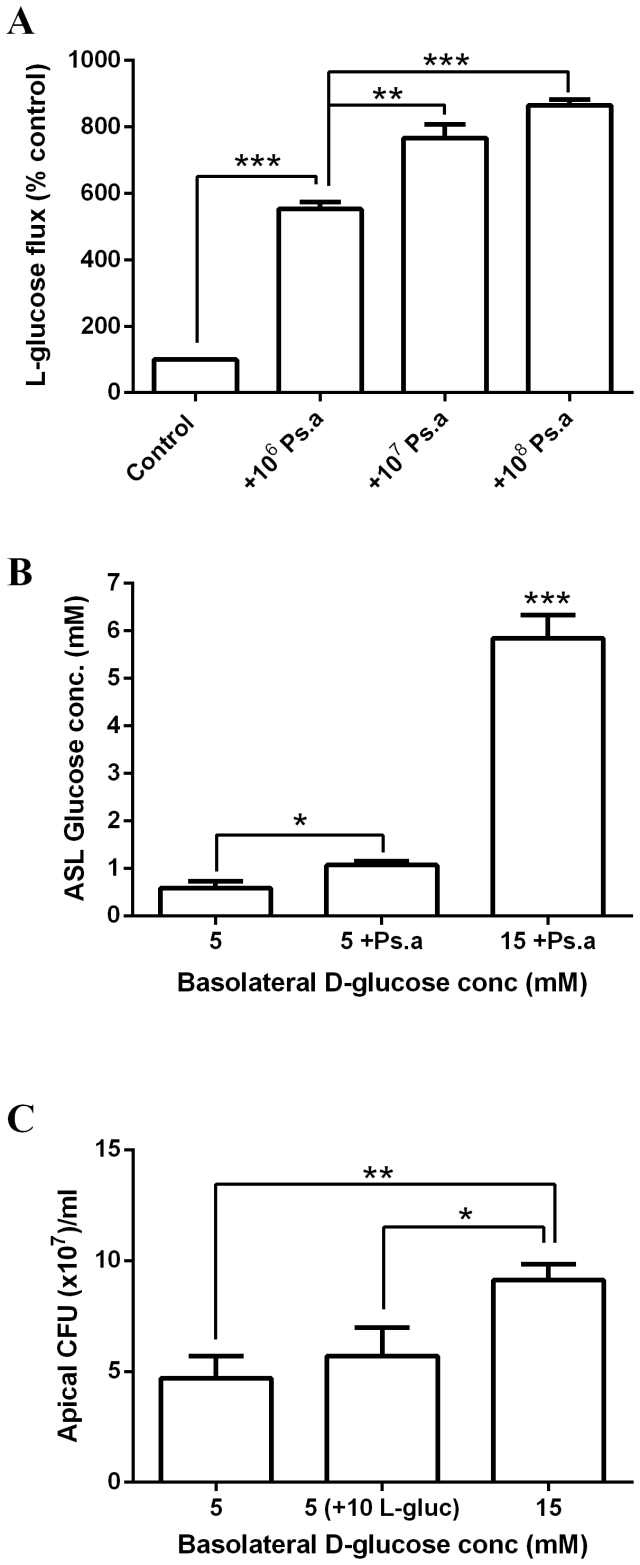
Apical *Ps. aeruginosa* addition increases glucose flux across Calu-3 airway epithelial monolayers, resulting in elevated ASL glucose and D-glucose dependent bacterial growth. A: Basolateral-to-apical paracellular L-glucose (^14^C-L-glucose) flux across untreated Calu-3 monolayers and monolayers pre-exposed to *Ps. aeruginosa* (1×10^6^, 1×10^7^ or 1×10^8^ CFU) for 7 hours. n = 3 in each group, obtained from 3 independent experiments. ** p<0.01, *** p<0.001. B: Glucose concentration of airway surface liquid (ASL) across Calu-3 airway epithelial monolayers after 24 hours, in the presence of 5 or 15 mM basolateral glucose, with or without *Ps. aeruginosa* filtrate (Ps. a). n = 3 in each group, obtained from 3 independent experiments. * p<0.05, *** p<0.001. C: *Ps. aeruginosa* growth over 7 hours across the apical surface of Calu-3 monolayers, in the presence of 5 mM D-glucose, 15 mM basolateral D-glucose or 5 mM D-glucose plus 10 mM L-glucose. n = 6 in each group, obtained from 3 independent experiments. * p<0.05, ** p<0.01.

The addition of *Ps. aeruginosa* to the apical surface of Calu-3 monolayers for 7 hours increased basolateral-to-apical L-glucose flux across the monolayers in a dose-dependent manner (p<0.001, n = 3; Figure 4A). To investigate whether increased flux resulted in elevated glucose in the ASL, *Ps. aeruginosa* filtrate was used to eliminate the possibility of bacterial glucose uptake affecting ASL concentration. *Ps. aeruginosa* filtrate increased the concentration of glucose present in the ASL from 0.6±0.1 mM to 1.1±0.1 mM (p<0.05, n = 3; Figure 4B). Increasing the basolateral concentration of D-glucose from 5 to 15 mM further increased ASL glucose concentrations from 1.1±0.1 mM to 5.8±0.8 mM (p<0.001, n = 3). When the growth of *Ps. aeruginosa* applied to the apical surface of Calu-3 monolayers was measured after 7 hours, increasing the basolateral concentration of D-glucose, but not L-glucose, resulted in an increase in growth from 4.7±1.0 CFU (x10^7^)/ml to 9.1±0.7 CFU (×10^7^)/ml (an increase of 94±19%; p<0.01, n = 6; Figure 4C), consistent with our observations in non-CF and CF HBE cells.

### Increasing basolateral glucose enhances apical *Ps. aeruginosa* growth on Calu-3 monolayers over and above the effects of CFTR-dependent secretions and mucus hyperviscosity

In order to gauge the potential importance of glucose-induced changes in bacterial growth in comparison to other hallmark abnormalities of the CF ASL, co-cultures were performed on Calu-3 airway epithelial cells. Calu-3 cultures are heterogeneous, consisting of both serous (fluid-secreting) and mucous (mucin-secreting) cell types, which produce CFTR-dependent secretions.

**Figure 5:**
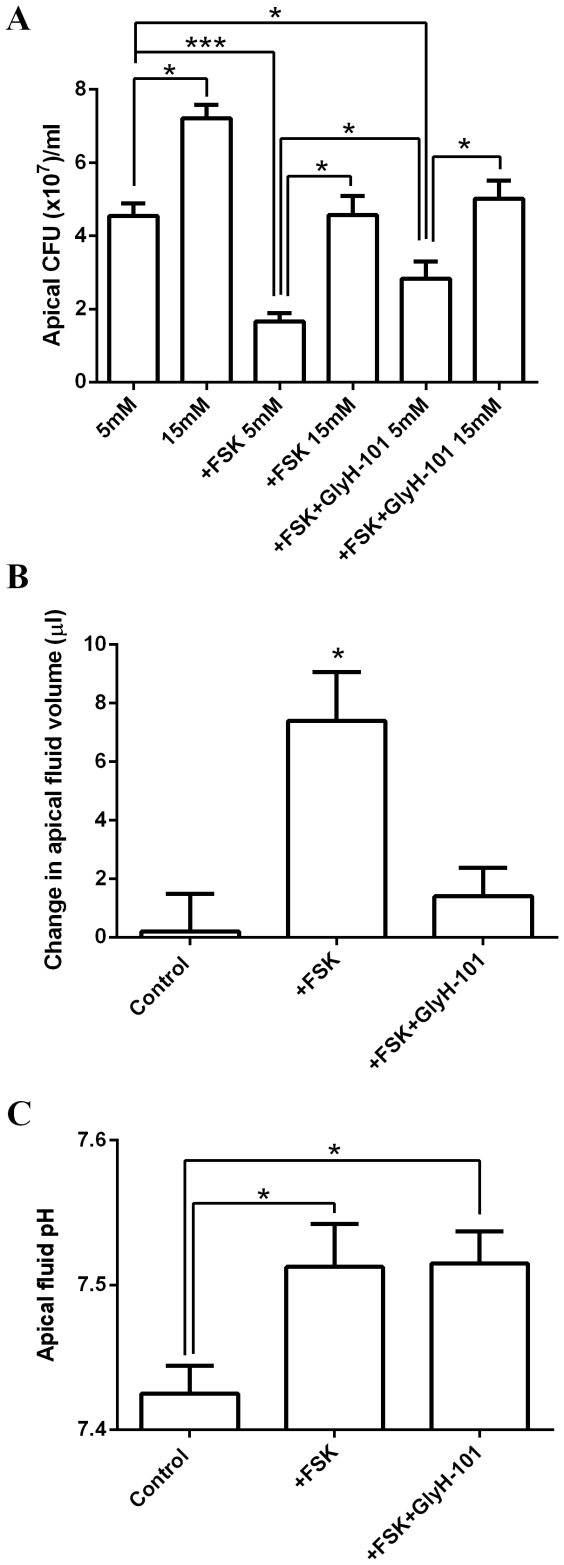
Increasing basolateral glucose enhances apical *Ps. aeruginosa* growth across Calu-3 monolayers over and above the effects of CFTR-dependent secretions. A: *Ps. aeruginosa* growth over 7 hours across the apical surface of untreated or forskolin-stimulated Calu-3 monolayers (±CFTR pore blocker GlyH-101), in the presence of 5 mM or 15 mM basolateral D-glucose. n = 6 in each group, obtained from 3 independent experiments. * p<0.05, *** p<0.001. B: Changes in the apical fluid volume ( µl) of *Ps*. aeruginosa-Calu-3 co-cultures over 7 hours (starting volume  = 100 µl). n = 6 in each group, obtained from 3 independent experiments. * p<0.05. C: Changes in the pH of apical fluid from *Ps. aeruginosa*-Calu-3 co-cultures over 7 hours. n = 6 in each group, obtained from 3 independent experiments. * p<0.05.

Addition of the cAMP-agonist forskolin, which stimulates CFTR-dependent fluid secretion from these cells [17], significantly reduced *Ps. aeruginosa* growth by 63±8% (in the presence of 5 mM basolateral glucose; p<0.001, n = 6; Figure 5A). However, *Ps. aeruginosa* growth remained higher in 15 mM glucose compared to 5 mM during forskolin stimulation (4.6±0.5 CFU (×10^7^)/ml in 15 mM glucose, compared to 1.7±0.2 CFU (×10^7^)/ml in 5 mM glucose; p<0.05, n = 6; Figure 5A). Forskolin-stimulated co-cultures increased the apical fluid volume by 7±1 µl after 7 hours (p<0.05, n = 6; Figure 5B) and the pH of the apical fluid increased from 7.43±0.02 to 7.51±0.03 pH units (p<0.05, n = 4; Figure 5C). The forskolin-mediated inhibition of *Ps. aeruginosa* growth could be partially overcome by the CFTR pore blocker GlyH-101 (p<0.05, n = 6; Figure 5A). However, the CFTR blocker had no effect on the 15 mM glucose-induced increase in *Ps. aeruginosa* growth. Although inhibiting CFTR abolished the forskolin-stimulated increase in apical fluid volume (p<0.05, n = 6; Figure 5B), it had no effect on the forskolin-induced alkalinisation (p>0.05, n = 4; Figure 5C). These data indicate that CFTR ion conductance is important for ASL antimicrobial activity but that elevation of basolateral glucose from 5 to 15 mM promotes *Ps. aeruginosa* growth irrespective of CFTR activity.

**Figure 6:**
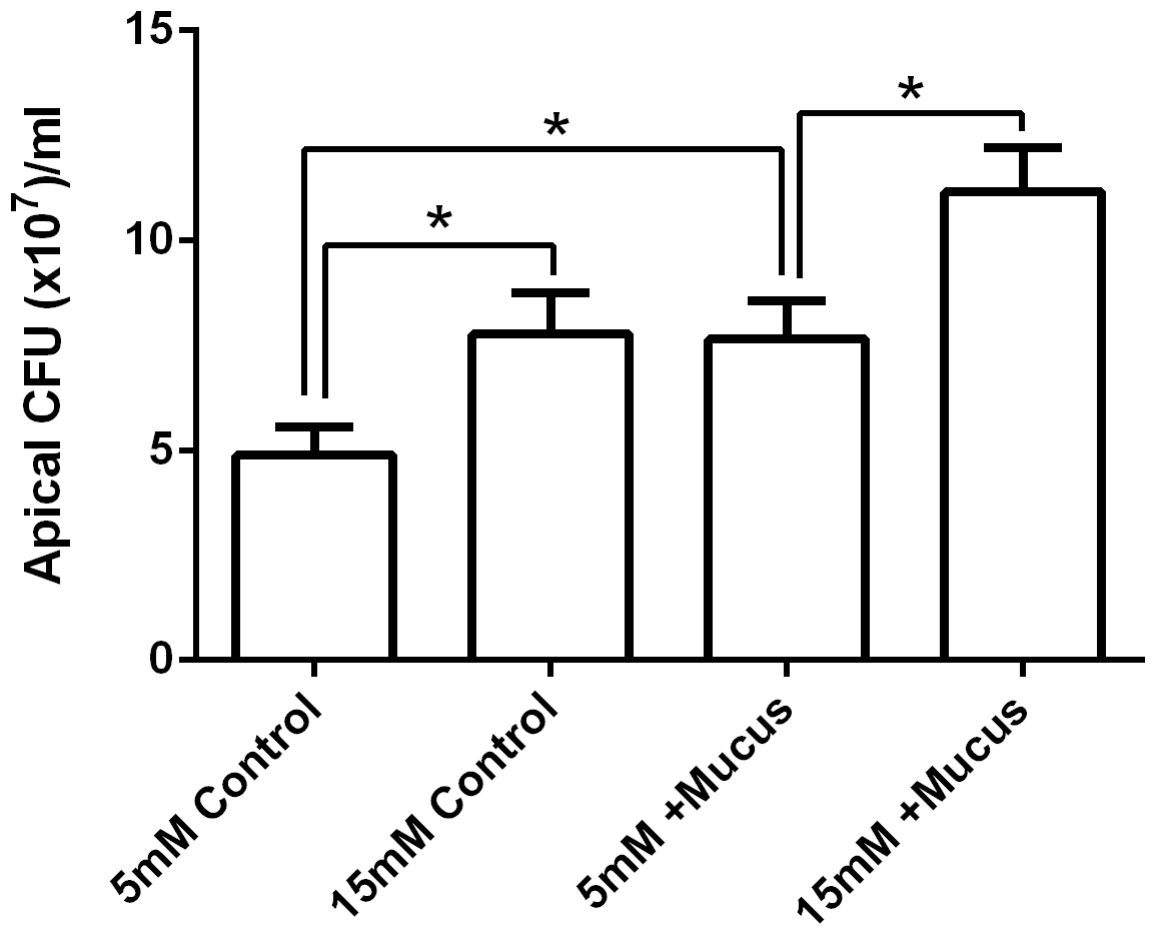
Increasing basolateral glucose enhances apical *Ps. aeruginosa* growth across Calu-3 monolayers over and above the effects of mucus hyperviscosity. *Ps. aeruginosa* growth over 7 hours across the apical surface of Calu-3 monolayers with or without a viscous mucus layer, in the presence of 5 mM or 15 mM basolateral D-glucose. n = 4 in each group, obtained from 4 independent experiments. * p<0.05.

Calu-3 monolayers grown at ALI generate a thick layer of mucus on the apical surface over time. The presence of this mucus layer increased *Ps. aeruginosa* CFU by 57±7%, compared to monolayers in which the mucus layer had been removed (in the presence of 5 mM basolateral glucose; p<0.05, n = 4; Figure 6). Importantly, elevating basolateral glucose increased *Ps. aeruginosa* CFU in the presence or absence of mucus (p<0.05, n = 4; Figure 6).

### Basolateral glucose alters airway epithelial monolayer transepithelial resistance

**Figure 7:**
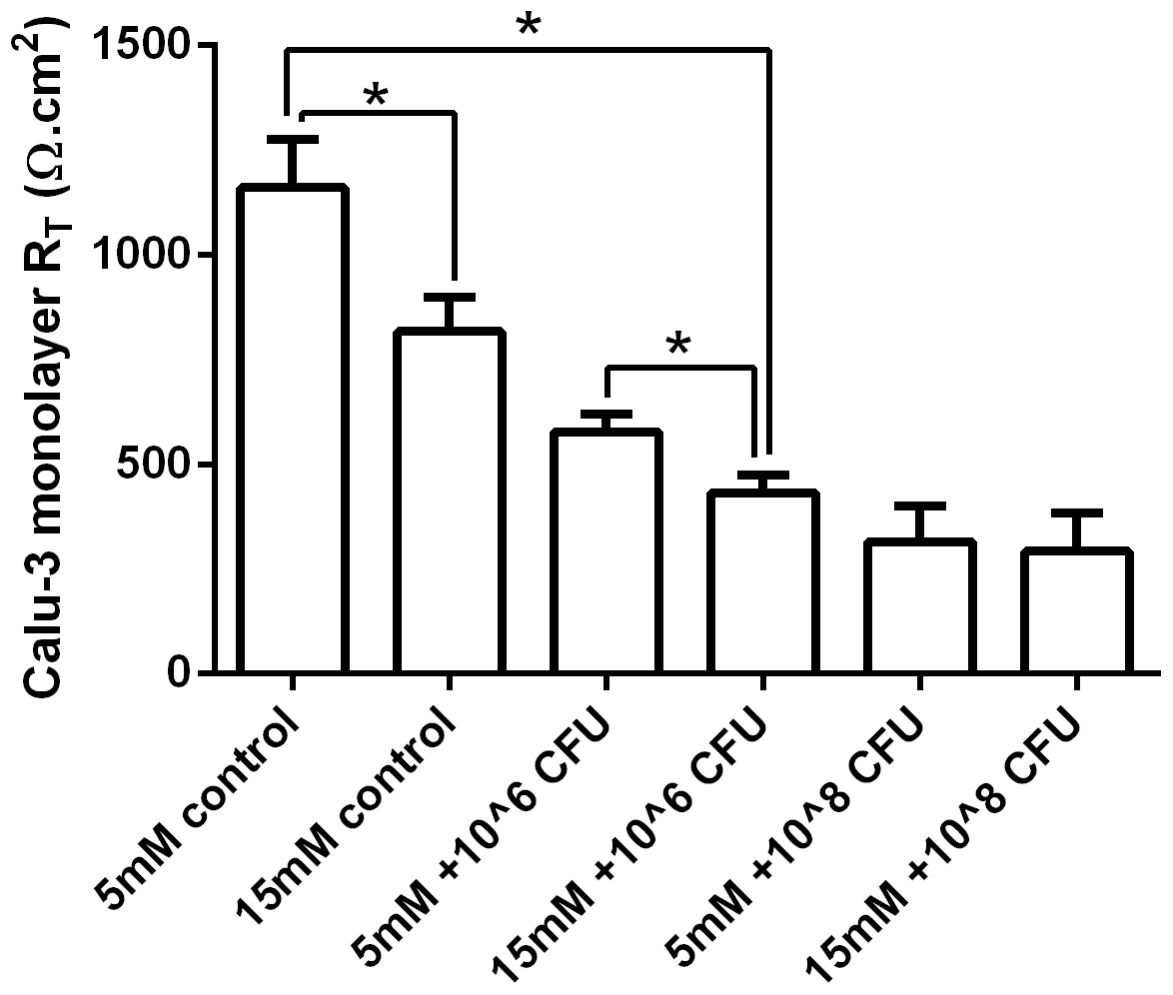
Basolateral glucose alters airway epithelial monolayer transepithelial resistance. Transepithelial resistance (R_T_) across untreated Calu-3 monolayers and Calu-3 monolayers co-cultured for 7 hours with 1×10^6^ or 1×10^8^ CFU of *Ps. aeruginosa* (Ps. a). n = 6 in each group, obtained from 3 independent experiments. * p<0.05.

Interestingly, we found that elevating the basolateral glucose concentration reduced transepithelial resistance (R_T_). Increasing basolateral glucose from 5 to 15 mM decreased Calu-3 monolayer R_T_ from 1160±115 Ω.cm^2^ to 816±83 Ω.cm^2^ (p<0.05, n = 6; Figure 7). A similar glucose-induced decrease in Calu-3 R_T_ was also observed after 7 hours of co-culture with 1×10^6^ CFU/cm^2^ *Ps. aeruginosa*, although this effect is likely to be, at least in part, due to the elevated growth of the bacteria in the presence of increased basolateral glucose. At higher bacteria inoculations, no significant difference in Calu-3 R_T_ was observed between glucose concentrations (p>0.05, n = 6).

## Discussion

We have previously shown that ASL glucose concentrations are elevated in people with chronic lung inflammation, including those with CF [6], [20]. ASL glucose concentrations are also increased by hyperglycaemia [6]. Diabetes mellitus is a common comorbidity with CF and the combination of lung inflammation and hyperglycaemia produces a further increase in ASL glucose concentrations [6].

Elevated ASL glucose concentrations are associated with an increased risk of respiratory infection and diabetes is a predisposing factor for acquisition of multiple antibiotic resistant *Ps. aeruginosa* and increased pulmonary exacerbations in people with CF [5]. The aims of this study were to use human airway epithelial cell culture models to explore mechanisms underlying the relationship between changes in ASL glucose and increased susceptibility to infection in CF.

We found that exposure to *Ps. aeruginosa* filtrate or live *Ps. aeruginosa*, decreased transepithelial resistance (R_T_) and increased paracellular glucose flux across human airway epithelial monolayers, consistent with our previous observations [8]. *Ps. aeruginosa*, in addition to cell-associated virulence factors such as LPS, pili and flagella, which can activate inflammatory responses via toll-like receptors (TLRs) [21], secrete an arsenal of factors that can damage the airway epithelium and alter cellular processes. Secreted proteases such as alkaline protease (AprA), endotoxins such as pyocyanin, ExoS, -T, -Y and rhamnolipids have all been shown to modify tight junction structure leading to decreased barrier function and contribute to the pathophysiology of CF disease [22], [23], [24]. Since the *Ps. aeruginosa* filtrate was boiled it is likely that its effects on the epithelium were due to heat-resistant secretions by the bacterium. Importantly, *Ps. aeruginosa* filtrate induced a greater increase in paracellular flux and reduction in R_T_ across CF than non-CF epithelia monolayers. This is consistent with previous observations that tight junctions are abnormal in CF airway epithelium and that CF epithelia display increased paracellular permeability to both solutes and ions in the presence of pro-inflammatory stimuli compared to normal airway cells [13], [25].

R_T_ was also reduced by raising basolateral glucose. It is not clear what mechanisms are involved in the response to hyperglycaemia, but it could involve changes in AMPK activity [26], pro-inflammatory cytokine release or glucose transporter activity [27]; all of which can modify tight junctions. In Calu-3 cells we measured ASL glucose concentration and found this to be increased by *Ps. aeruginosa* filtrate and further by elevation of basolateral glucose concentrations. Thus, our experimental conditions mimic the state of the airways in patients with CFRD, where inflammation, *Ps. aeruginosa* infection and hyperglycaemia could all increase paracellular permeability and drive movement of glucose into the ASL.

Glucose is removed from ASL by apical glucose transport. We have previously shown in human H441 airway cells that pro-inflammatory stimuli increase GLUT2 and GLUT10 abundance and apical glucose uptake [10]. However, this was insufficient to prevent elevation of ASL glucose, as paracellular glucose flux exceeded uptake. Similarly, in the current study, apical glucose uptake was increased in both non-CF and CF epithelial cell monolayers by exposure to *Ps. aeruginosa* filtrate. Interestingly, there was no difference in the stimulated apical glucose uptake between the two cell types, despite the much greater increase in paracellular glucose flux across the CF monolayers. Thus, we propose that under pro-inflammatory conditions, glucose uptake is not sufficient to compensate the increased flux of glucose into ASL, particularly in CF epithelial cells.

Elevation of basolateral D-glucose evoked an increase in apical *Ps. aeruginosa* growth in all airway epithelial co-culture models investigated in this study. *Ps. aeruginosa* utilises glucose as a growth substrate [20] and so increased growth appears to be a direct effect of increased availability of this nutrient. In support of this hypothesis, increased basolateral L-glucose, which diffuses across the tight junctions in the same way as D-glucose but is not commonly metabolised by bacteria [28], did not stimulate the growth of apical *Ps. aeruginosa*. Furthermore, the growth of *Ps. aeruginosa* was greater in the lungs of hyperglycemic than of normoglycemic mice, but there was no enhanced growth of a mutant strain of *Ps. aeruginosa* (PA01) with a defect in a gene (edd) required for catabolism of glucose [9].

Elevation of basolateral glucose from 5 to 15 mM had a greater effect on apical growth of *Ps. aeruginosa* on CF compared to non-CF monolayers. We speculate that the combination of increased paracellular permeability in CF monolayers with an elevated basolateral-to-apical glucose gradient further increased the availability of glucose to the bacteria, enhancing growth. In laboratory culture we have previously shown a dose-dependent effect of glucose, at concentrations found in airway surface liquid, on bacterial growth [20].

Currently, there is much research into how the lack of CFTR function in CF might affect ASL volume, composition and pH to alter bacterial growth [4], [29]. Impaired fluid secretion and hyperabsorption in CF result in thick viscous mucus, which is also thought to promote bacterial growth. Consistent with this notion, we found that the presence of a thick layer of mucus in Calu-3 cells increased the growth of *Ps. aeruginosa*. In Calu-3 cells, we found that forskolin (a cAMP agonist and activator of CFTR) increased fluid secretion, elevated pH and reduced bacterial growth. The CFTR pore blocker (GlyH-101) inhibited forskolin-induced fluid secretion, but did not reduce the forskolin-induced ASL alkalinisation. Under these circumstances bacterial growth was partially restored, but not to levels observed under control conditions. This indicates that forskolin stimulates both CFTR-dependent and -independent effects on bacterial growth. The forskolin-induced CFTR-dependent inhibition of *Ps. aeruginosa* growth was associated with increased ASL fluid volume. Interestingly, in support of this finding, reduced CFTR expression and depletion of ASL fluid volume has been proposed to produce a more suitable environment for *Ps. aeruginosa* colonisation in the lung [30], [31]. The forskolin-induced elevation of ASL pH was CFTR-independent. These findings are consistent with previous studies of Calu-3 fluid secretions, which identified the anion exchanger pendrin (also activated by elevation of cAMP), and not CFTR, as a key mediator of bicarbonate secretion from these cells [17]. Our data show that elevation of ASL pH was associated with decreased bacterial growth and elevated ASL pH is known to increase the activity of pH-sensitive antimicrobials [4].

Crucially, even in the context of these well-recognised CF-related abnormalities, elevation of basolateral glucose had a marked additional stimulatory effect on bacterial growth. In people with CF, the development of diabetes is associated with accelerated pulmonary decline, increased risk of infection with multiple antibiotic-resistant *Ps. aeruginosa* [5] and increased pulmonary exacerbations [32]. These data provide evidence that increased glucose leak into ASL as a consequence of rising blood glucose and worsening lung inflammation could provide an additional stimulus respiratory infection and augment the effects of other CF-related abnormalities.

## Conclusions

We have shown that elevation of basolateral glucose promotes the apical growth of *Ps. aeruginosa* on CF airway epithelial monolayers more than non-CF monolayers. *Ps. aeruginosa* secretions induced an elevated glucose leak across CF airway epithelial monolayers compared to non-CF monolayers which we propose increases ASL glucose availability for bacterial growth. In addition, elevating basolateral glucose increased *Ps. aeruginosa* growth over and above the effects of CFTR-dependent fluid secretions and mucus in Calu-3 epithelial-bacterial co-cultures. Together these studies highlight the potential importance of glucose in promoting *Ps. aeruginosa* growth and respiratory infection in CF and CF-related diabetes and identify potential new targets for the prevention and treatment of CF pulmonary infection.
